# I’m still quite young: women’s lived experience of precocious or premature menopause: a qualitative study among Egyptian women

**DOI:** 10.1186/s12912-025-03133-6

**Published:** 2025-05-28

**Authors:** Yasmine M. Osman, Hend Reda Ali El-Kest, Majed Awad Alanazi, Mostafa Shaban

**Affiliations:** 1 Department of Obstetrics and Gynecology Nursing, Faculty of Nursing, Zagazig University, Zagazig, Egypt; 2 Faculty of Nursing, Tanta University, Tanta, Egypt; 3 Medical Surgical Nursing Department, College of Nursing, Jouf University, Sakaka, Saudi Arabia; 4 Present Address: College of Nursing, Jouf University, Sakaka, Saudi Arabia

**Keywords:** Precocious menopause, Women’s health, Nursing support, Cultural stigma, Psychosocial care, Qualitative study

## Abstract

**Background:**

Precocious menopause, the cessation of ovarian function before age 40, presents complex physical, emotional, and socio-cultural challenges. In settings where fertility is central to a woman’s identity, early menopause can lead to stigma, psychological distress, and limited healthcare engagement. Nurses play a pivotal role in supporting affected women through education and empathetic care.

**Aim:**

This study explored the lived experiences of Egyptian women diagnosed with precocious menopause, with a focus on physical and emotional challenges, cultural and familial dynamics, coping mechanisms, and interactions with healthcare providers—particularly nurses.

**Methods:**

A qualitative descriptive design was employed. Fifteen women diagnosed with precocious menopause were recruited through purposive sampling from reproductive health clinics in Tanta city and surrounding areas in the Nile Delta region. Data were collected through semi-structured, in-depth interviews and analyzed using thematic content analysis.

**Results:**

Participants reported significant disruption to self-identity and emotional well-being, compounded by cultural stigma and family pressures. Coping strategies included spiritual beliefs, peer support, and self-advocacy. Experiences with healthcare providers were mixed; nurses emerged as key figures in providing psychosocial support and education.

**Conclusion:**

Findings underscore the need for culturally sensitive, nurse-led interventions to address the unique needs of women with precocious menopause. Enhancing nursing education and integrating psychosocial care into reproductive health services can improve health outcomes and quality of life.

**Clinical trial number:**

Not applicable.

**Supplementary Information:**

The online version contains supplementary material available at 10.1186/s12912-025-03133-6.

## Introduction

Menopause is typically defined as the permanent cessation of menstruation due to the loss of ovarian follicular activity, occurring between the ages of 45 and 54 years [1]. Although a natural biological transition, menopause is often accompanied by considerable physical and psychological challenges [2]. When menopause occurs prematurely—before the age of 40—it is referred to as precocious or premature menopause (PM). This condition, also known as premature ovarian failure (POF), dysfunction (POD), or insufficiency (POI), is characterized by the absence of menstruation for 12 consecutive months prior to age 40 [3, 4].

Globally, research has identified varying rates of spontaneous premature menopause and POI. A recent meta-analysis involving over 150,000 women estimated that 3.7% experience spontaneous premature menopause, and 12.2% experience early menopause [5]. Regional data show prevalence rates of 3.2% in Iran [6], 10.9% in China, and 20.2% in India for early menopause [6]. In Egypt, Demographic and Health Survey data report that 2.2% of women experience premature menopause, with 6.27% undergoing early menopause—figures notably higher than the global average [7].

PM may arise spontaneously or result from medical interventions such as chemotherapy, radiation, or surgical removal of the ovaries [8]. A range of factors—including genetic, hormonal, developmental, environmental, and lifestyle influences—can affect the timing of menopause [6]. Women with PM face increased risks for chronic health conditions, including cardiovascular disease, osteoporosis, cognitive decline, type 2 diabetes, and sexual dysfunction [9–13].

Beyond physical symptoms, PM has a substantial emotional and social impact. Women often struggle with disrupted life plans, including postponed or unfulfilled reproductive goals. This loss is compounded by prevailing social norms that link womanhood with fertility. Women may face judgment or marginalization, which intensifies their emotional strain [14]. Although symptoms of PM resemble those of natural menopause, the experiences and needs of younger women differ significantly [15]. Research shows that PM is associated with heightened levels of depression, reduced self-esteem, and adverse effects on sexuality, identity, and quality of life [16, 17]. The experience may also challenge women’s sense of self and their roles within relationships and society [18].

Despite these challenges, many women lack adequate knowledge about PM, treatment options, and lifestyle adaptations. Healthcare professionals—especially nurses—play an essential role in bridging this gap through education, counseling, and support. In Egypt, where menopause is often underdiscussed and fertility holds high cultural value, PM carries unique implications. Young women with PM may feel disconnected from their peers and affected by stereotypes portraying menopause as an older woman’s condition [19]. These cultural perceptions further complicate the emotional burden of early menopause.

While existing literature has largely focused on quantitative assessments or secondary data such as meta-analyses [20, 21], there is a notable gap in understanding the lived experiences of Egyptian women with PM. Given cultural differences in how menopause is perceived and experienced, qualitative methods are valuable for capturing nuanced, context-specific insights. This study therefore seeks to explore how Egyptian women navigate the physical, emotional, social, and cultural dimensions of precocious menopause, and how their experiences shape their coping and support needs.

### Aim of the study

This study aims to explore the lived experiences of Egyptian women diagnosed with precocious menopause, with a primary focus on the personal, emotional, and socio-cultural implications of early ovarian failure. It seeks to understand how these women perceive and navigate their condition within the context of prevailing cultural norms, gender expectations, and family dynamics. Additionally, the study examines their interactions with healthcare providers—particularly nurses—to identify gaps in support and opportunities for culturally sensitive care. The insights generated will contribute to developing tailored interventions and nursing strategies that address the unique psychosocial and cultural needs of this population.

### Research questions

1. How do Egyptian women diagnosed with precocious menopause experience and interpret the personal and socio-cultural challenges associated with their condition?
2. In what ways do cultural beliefs, family expectations, and societal norms influence their coping strategies and self-perception?
3. How do these women describe their interactions with healthcare providers, particularly nurses, and what are their perceptions of the support they receive?

## Methods

### Study design

The study used a qualitative descriptive approach, based on naturalism and constructivism, to investigate the lived experiences of Egyptian women diagnosed with early menopause. This methodology was chosen to capture the depth of participants’ personal experiences, which provide valuable insights into the biology and socio-cultural aspects of early ovarian failure. The study complied with the Standards for Reporting Qualitative Research (SRQR) requirements, guaranteeing methodological rigour and openness throughout the research process [22]. The research seeks to uncover the intricate obstacles women encounter and how cultural and family factors influence their coping techniques by enabling them to articulate their experiences in their own words.

### Study setting

The research was conducted in Tanta city, located in Egypt’s Nile Delta region, and its surrounding areas, specifically including El-Mahalla El-Kubra and Kafr El-Zayat within the Gharbia Governorate. Data were collected from a range of reproductive health clinics and community centers that serve women from various socio-economic and cultural backgrounds. These facilities were selected for their accessibility and their comprehensive services in gynecology, reproductive counseling, and hormonal health. The urban and semi-urban mix offered a rich setting to explore the intersection of modern medical care and traditional beliefs.

### Participants

A purposive sampling technique was employed to recruit 15 Egyptian women diagnosed with precocious menopause prior to the age of 40. Participants were required to be native Arabic speakers with a confirmed diagnosis, ensuring they could articulate their experiences fully and accurately in their own language. Women from diverse socio-economic statuses, educational backgrounds, and marital statuses were included to encompass a wide range of experiences. Recruitment occurred via local reproductive health clinics, community health centres, and support networks utilised by women for guidance on reproductive matters. The participants’ age range was 28 to 39 years, representing diverse life stages and social roles, thereby offering valuable insights into the impact of the diagnosis on different aspects of their lives. Women with cognitive impairments or those lacking a formal diagnosis were excluded to preserve the integrity and depth of the data.

### Pilot interview

Prior to formal data collection, a pilot interview was conducted with one eligible participant to assess the clarity, sensitivity, and flow of the interview guide. Based on feedback, minor adjustments were made to improve question wording and sequencing. The data from the pilot were not included in the final analysis.

### Data collection

Data were collected over a four-month period from january to febuary 2025, through face-to-face, semi-structured, in-depth interviews, utilising a semi-structured interview protocol developed and tailored for this study. The interview guide was developed following a thorough literature review and expert consultations in women’s health and qualitative research. It encompasses essential topics, including initial emotional responses to diagnosis, physical and psychological symptoms, effects on self-identity, familial and marital relationships, and culturally influenced coping strategies.

Interviews occurred in private rooms within healthcare facilities, providing a secure and confidential environment for participants to share their experiences openly. The duration of each interview was approximately 45 to 60 min, and audio recordings were made with the participants’ consent. In addition to the recordings, comprehensive field notes were documented to capture non-verbal cues, environmental context, and any spontaneous reflections that could enhance the data. The utilisation of Arabic as the communication medium facilitated participants’ natural expression and preserved cultural nuances in their narratives.

Follow-up interviews were conducted with several participants to enhance the understanding of emerging themes. The follow-up sessions enabled the researcher to elucidate previous responses, explore newly emerging concepts, and guarantee that the data collection process was iterative and thorough. Alongside the interviews, pertinent documents, including patient education materials on menopause and local healthcare guidelines, were examined to enhance context and triangulate the data gathered from participants.

### Credibility of the study

Various strategies were employed to establish the credibility and trustworthiness of the findings. Prolonged engagement was attained through extensive interaction with each participant during the initial and follow-up interviews. This interaction facilitated the development of rapport and a more profound comprehension of the personal and cultural factors affecting their experiences. The researcher kept a reflective journal during the study to record personal insights, methodological choices, and potential biases, ensuring transparency in the research process.

Data triangulation was achieved through the collection of information from various sources, such as audio-recorded interviews, comprehensive field notes, and document analyses. This triangulation facilitated the cross-verification of themes identified in the interviews, thereby strengthening the overall robustness of the findings. Furthermore, regular peer debriefing sessions were conducted with colleagues who possess expertise in qualitative research. The sessions served as an external evaluation of the coding process, facilitating the refinement of emerging themes by questioning initial interpretations and presenting alternative viewpoints.

Member checking served as a crucial element in establishing the study’s credibility. Participants reviewed a summary of the key themes identified from their interviews, enabling them to confirm or clarify the interpretations of their experiences. The collaborative feedback process ensured that the findings aligned with participants’ lived experiences and reduced the likelihood of misinterpretations.

### Data analysis

Data were analyzed using thematic content analysis, guided by Braun and Clarke’s six-step framework. Audio recordings were transcribed verbatim in Arabic by a bilingual researcher proficient in medical and cultural terminology. Transcripts were reviewed multiple times for immersion, and initial codes were manually developed using Microsoft Excel to track, cluster, and compare data across cases.

Codes were then grouped into categories and broader themes, reflecting shared patterns in the women’s lived experiences. Examples of key codes included: *“initial shock*,*” “loss of femininity*,*” “family pressure*,*”* and *“religious acceptance.”* A thematic map was developed to illustrate connections across categories.

To enhance trustworthiness, a second qualitative researcher independently reviewed 5 out of the 15 transcripts using the initial coding framework. Discrepancies were discussed in collaborative sessions, and consensus was achieved through peer debriefing and refinement of the codebook. This approach supported inter-coder agreement and thematic clarity.

Although the analysis followed an inductive process, the interpretation of themes was informed by social constructivist theory, emphasizing the influence of cultural and interpersonal meaning-making on the participants’ experiences.

In the initial coding phase, key statements, phrases, and reflections were identified. The initial codes were recorded with comprehensive notes that documented the context and possible significance of each segment. Codes such as “initial shock and denial,” “fear of infertility,” “sense of lost femininity,” “family-related stigma,” and “reliance on spiritual beliefs” were identified from the data. The codes were organised into broader categories according to shared characteristics, establishing a foundation for the emergence of themes.

Examples of initial codes identified included:

- **Emotional Turmoil**: Expressions of shock, denial, grief, and anxiety following the diagnosis.
- **Loss of Identity**: Narratives reflecting a perceived loss of femininity and self-worth.
- **Fertility Concerns**: Fears related to the inability to conceive and the social implications of infertility.
- **Cultural and Social Pressures**: Accounts of societal expectations, stigma, and familial pressures regarding traditional roles and reproduction.
- **Coping Strategies**: References to the use of religious faith, community support, and personal resilience as mechanisms to manage the condition.

### Ethical considerations

The Institutional Review Board (IRB) of the Faculty of Nursing, Tanta University, Egypt, provided ethical permission for this research (IRB No. 2025-1-586). Before their involvement, all women were provided with comprehensive information on the study’s aims, methodologies, possible dangers, and expected advantages. Informed permission was secured from each participant, ensuring that their involvement was completely voluntary and that they may withdraw at any moment without facing any negative repercussions.

To protect participants’ anonymity, all interviews were held in private environments, and the audio recordings and transcripts were anonymised by assigning unique identities to each participant. Data were securely kept on encrypted devices, with access limited only to the study team. The research adhered to the ethical standards established by the Declaration of Helsinki, assuring the strict protection of participants’ rights, dignity, and privacy throughout the study.

## Results 

This study involved women diagnosed with precocious menopause, aged 27 to 41 years (Table 1). This age variation offers insights into the impact of early ovarian failure on women across various life stages, from their late twenties, when fertility expectations remain significant, to their late thirties and early forties, when additional social and psychological issues may arise.

The participants exhibited a range of marital statuses, including married, single, and divorced women, facilitating a comprehensive analysis of how relational dynamics and societal expectations affect the experience of precocious menopause. The participants exhibited diverse educational backgrounds, with some possessing bachelor’s or master’s degrees and others holding diplomas, indicating a spectrum of knowledge and potential access to medical information regarding their condition.

The duration since diagnosis varied from 2 to 9 years, encompassing women who were newly adjusting to their diagnosis and those who had been managing the condition for an extended period. The combination of experiences enhanced the study by documenting both immediate responses and enduring adaptation strategies.

**Table 1:** Demographic charchtersitics of the participants

Participant	Age (Years)	Marital status	Education level	Years since diagnosis
P1	29	Married	Bachelor Degree	5
P2	35	Single	Master Degree	7
P3	32	Married	Bachelor Degree	4
P4	38	Married	Diploma	6
P5	40	Divorced	Bachelor Degree	8
P6	28	Single	Diploma	3
P7	37	Married	Bachelor Degree	5
P8	33	Single	Bachelor Degree	4
P9	31	Divorced	Diploma	6
P10	36	Married	Master Degree	9
P11	39	Married	Bachelor Degree	5
P12	30	Single	Diploma	3
P13	34	Married	Bachelor Degree	7
P14	41	Married	Bachelor Degree	8
P15	27	Single	Diploma	2

## Thematic analysis of women’s experiences of precocious menopause

The following part provides a detailed account of the themes and subthemes emerging from the qualitative analysis of Egyptian women’s experiences with precocious menopause (Table 2). By integrating direct quotes from participants, observational notes, and relevant document insights, the analysis reveals the multifaceted nature of the experience—ranging from the physical and psychological challenges to the socio-cultural pressures, adaptive coping strategies, and healthcare-related issues. The themes are organized into five main categories, highlighting the complexities and nuances of navigating early menopause within a unique cultural context.

### Physical and psychological impact of precocious menopause

#### Symptom burden and physical changes

Women diagnosed with precocious menopause frequently described experiencing a sudden and overwhelming array of physical symptoms that disrupted their daily routines and overall well-being. Many participants expressed that these changes were unexpected, as they had associated menopause with later stages of life. Participant P3 explained, *“I was completely unprepared for the night sweats and the constant fatigue. I thought menopause was something that happened to women much older than me.”* Similarly, Participant P7 shared, *“My body felt like it aged overnight. I had joint pain*,* hot flashes*,* and my skin started changing. It was terrifying.”*

Observational data supported these accounts, as several participants appeared visibly fatigued and fanned themselves frequently during interviews, even in air-conditioned rooms. Document reviews also revealed multiple instances where women had sought medical attention for persistent headaches, dizziness, and sleep disturbances before receiving a confirmed diagnosis of precocious menopause. Many described their symptoms as debilitating, impacting not only their energy levels but also their ability to work and engage in social activities.

Another common challenge was the rapid onset of hormonal imbalances, which participants often compared to an emotional and physical rollercoaster. Participant P10 expressed frustration over how unpredictable her body had become: *“One day I would feel fine*,* and the next*,* I would wake up drenched in sweat*,* completely exhausted. I never knew what to expect*,* and that made it so hard to plan anything.”* This sense of unpredictability contributed to increased stress, as women struggled to regain control over their health and daily functioning.

#### Emotional and mental health challenges

Beyond the physical changes, the psychological impact of precocious menopause was profound, with many participants describing feelings of grief, anxiety, and self-doubt. Several women compared their experience to a sense of mourning—grieving not only the loss of their fertility but also the future they had envisioned for themselves. Participant P5 explained, *“It felt like my body had betrayed me. I had always dreamed of having children*,* and suddenly*,* that possibility was taken away. It was devastating.”*

Many participants also reported experiencing intense anxiety and depression following their diagnosis. The abrupt hormonal changes were often accompanied by mood swings, feelings of hopelessness, and difficulty concentrating. Participant P1 described the emotional turmoil: *“One moment*,* I would be fine*,* and the next*,* I would feel like crying for no reason. It made me feel out of control.”* These psychological effects were exacerbated by the lack of awareness and social support surrounding precocious menopause. Women frequently felt misunderstood, with family members and even healthcare providers downplaying their distress.

Observational data indicated that participants often hesitated when discussing their emotional struggles, as if uncertain whether their feelings were valid. Some participants admitted that they initially dismissed their own distress, believing they had no right to grieve something they were told was “just a medical condition.” Document reviews also revealed that psychological symptoms were often underreported in medical records, with limited references to mental health referrals or counseling.

A recurring theme among participants was the challenge of feeling “out of sync” with their peers. While friends and colleagues of similar age were focused on career growth, marriage, or starting families, women experiencing precocious menopause felt increasingly isolated. Participant P9 shared, *“I felt like I was living in a different world from my friends. They were talking about their pregnancies*,* and I was dealing with menopause symptoms. It was a lonely place to be.”*

The psychological distress was further compounded by feelings of diminished femininity and attractiveness. Several women reported struggling with self-esteem and body image issues, particularly in relation to changes in weight, skin texture, and libido. Participant P6 noted, *“I didn’t feel like myself anymore. My body looked different*,* and I was constantly tired. I started avoiding mirrors because I didn’t like what I saw.”* These concerns, combined with societal pressures surrounding femininity and youth, added an additional layer of emotional burden.

### Socio-Cultural influences on the experience

#### Cultural stigma and societal expectations

Cultural perceptions of womanhood and fertility deeply influenced how participants experienced precocious menopause. In many cases, societal expectations surrounding a woman’s ability to conceive played a significant role in shaping their emotional response to the diagnosis. One participant expressed, “In our culture, a woman is expected to have children. When you can’t, people look at you differently, as if something is wrong with you.” This sense of stigma was reinforced by the reactions of extended family members and community figures, who often viewed early menopause as an unnatural or shameful condition.

Observational data confirmed that discussions about fertility were met with visible discomfort among participants, with some avoiding eye contact or hesitating before speaking. Several participants shared experiences of being subjected to unsolicited advice or even blame for their condition. One woman recalled, “A relative told me I must have done something wrong in my past to deserve this. As if it were a punishment.” Such narratives highlight the deep-seated cultural beliefs that link reproductive capacity to personal virtue, making it difficult for women to openly discuss their condition or seek support.

Medical records and healthcare notes further illustrated the reluctance of some women to disclose their diagnosis. In several instances, participants reported delaying or avoiding doctor visits due to fear of being labeled as infertile. The cultural framing of menopause as the “end of a woman’s youth” contributed to these anxieties, leaving many women struggling with feelings of inadequacy and low self-esteem.

#### Family and marital dynamics

The role of family in shaping a woman’s experience of precocious menopause was profound. While some participants described supportive spouses and relatives, many encountered challenges in their personal relationships due to their condition. One participant shared, “My husband became distant after my diagnosis. He didn’t say anything directly, but I could feel the difference—like I had become less valuable to him.” Marital strain was a recurring theme in participants’ narratives, particularly in cases where fertility expectations were unmet.

Several women reported that their families, especially in-laws, placed pressure on them to continue seeking fertility treatments despite the biological limitations imposed by menopause. “My mother-in-law kept insisting I try different herbal remedies or go to a spiritual healer. She couldn’t accept that I wasn’t going to have children,” explained one participant. This family pressure often led to emotional distress and internal conflict, as women struggled to balance personal acceptance with the need to meet societal and familial expectations.

Conversely, a few participants described having partners who stood by them, providing emotional and practical support. One woman reflected, “My husband told me that our marriage was about more than children. That made all the difference in how I coped with everything.” These positive accounts underscore the critical role of familial support in easing the psychological burden of early menopause.

Healthcare providers also noted the influence of family members on treatment decisions. In multiple cases, women delayed seeking hormone replacement therapy (HRT) or other medical interventions due to the skepticism of their spouses or parents. One participant explained, “My husband was afraid the medications would have side effects, so he didn’t want me to take them. I had to convince him before I could start treatment.” This dynamic suggests that family approval plays a significant role in shaping women’s healthcare choices, highlighting the need for broader education and awareness campaigns targeting not only patients but also their families.

### Coping strategies and adaptation

#### Reliance on spiritual and religious beliefs

For many women experiencing precocious menopause, spirituality and religious beliefs served as a crucial coping mechanism, helping them to make sense of their condition and find emotional solace. Several participants described how their faith played a central role in their ability to endure the psychological and physical challenges of early menopause. Participant P3 shared, *“At first*,* I felt like I was being punished*,* but over time*,* I started to see it as a test of faith. I pray more now*,* and it helps me accept what has happened.”* This sentiment was echoed by other women, who viewed their condition as part of a divine plan, fostering resilience through religious devotion.

Observational data confirmed the significance of faith-based coping, as many participants referenced religious phrases and expressions of gratitude despite their struggles. During interviews, some participants clutched prayer beads or mentioned increased participation in religious gatherings as a source of strength. Participant P8 explained, *“Whenever I feel overwhelmed*,* I turn to the Quran. It reminds me that everything happens for a reason*,* and that eases my heart.”* This spiritual perspective not only provided emotional relief but also shaped their approach to healthcare, with some women preferring natural remedies or trusting in divine healing over medical interventions.

However, reliance on spirituality was a double-edged sword for some participants. While it provided comfort, it sometimes discouraged them from seeking medical treatment or psychological support. Participant P5 admitted, *“I used to think that I should just endure this and not question it. But eventually*,* I realized that I needed help and that faith and medicine could go hand in hand.”* These findings highlight the importance of healthcare professionals integrating culturally sensitive discussions about faith while encouraging women to seek comprehensive medical care.

#### Seeking social and peer support

In addition to religious coping, many participants emphasized the value of social and peer support in navigating the challenges of early menopause. Women who had access to understanding friends, family, or online communities reported feeling less isolated and more empowered. Participant P6 noted, *“Talking to other women who have gone through the same thing was a game changer for me. Before that*,* I thought I was completely alone in this.”* Online support groups, in particular, were identified as a safe space for sharing experiences, exchanging practical advice, and receiving emotional validation.

Observational data reinforced this theme, as participants who engaged with peer networks often displayed more confidence in discussing their condition and exploring management strategies. Many reported that hearing the experiences of others helped them reframe their diagnosis and find a sense of belonging. Participant P10 expressed, *“At first*,* I kept everything to myself. I didn’t even tell my best friend. But when I joined a support group*,* I realized that so many women were struggling with the same emotions. It made me feel normal again.”*

However, not all women had access to supportive social circles. Some participants reported experiencing judgment or dismissiveness from friends and family, making their adjustment to early menopause even more difficult. Participant P7 shared, *“When I tried to open up*,* some people just brushed it off*,* saying*,* ‘It’s not a big deal*,* you’ll be fine.’ But for me*,* it was a huge deal.”* This lack of understanding sometimes forced women to seek support outside their immediate social networks, reinforcing the need for awareness campaigns and more structured support groups for women with precocious menopause.

### Personal growth and empowerment

#### Reframing identity and life perspectives

For many women diagnosed with precocious menopause, the experience initially brought feelings of loss and uncertainty. However, over time, some participants described a shift in perspective, where they began to redefine their identity beyond their reproductive capabilities. One participant reflected, “At first, I felt like I had lost something fundamental. But with time, I realized that being a woman is not just about having children—it’s about so much more.”

This transformation often involved a re-evaluation of personal goals and aspirations. Several women found new ways to find fulfillment, whether through career advancement, personal development, or engaging in social and community activities. Another participant shared, “I started focusing on my passions, things I had neglected before. I learned that my worth is not tied to my ability to conceive.”

Observational insights revealed that many participants who had undergone this shift in mindset appeared more confident and engaged in their daily lives. Some had taken proactive steps to mentor and support younger women going through similar experiences, demonstrating a sense of empowerment that came from embracing their new reality.

Clinical notes also highlighted how psychological resilience played a role in helping women adapt to the emotional challenges of early menopause. Healthcare providers noted that women who received counseling or engaged in peer support networks displayed a greater sense of acceptance and well-being over time Table 2.

#### Resilience and self-advocacy

Despite the challenges associated with precocious menopause, many participants described how the experience strengthened their resilience and sense of self-advocacy. Women became more proactive in seeking medical information, pushing for better healthcare services, and educating themselves about their condition. One woman stated, “Before my diagnosis, I never questioned doctors. Now, I make sure to ask the right questions and advocate for myself in every medical appointment.”

Participants also emphasized the importance of self-care and prioritizing their own well-being. One woman remarked, “I learned to listen to my body, to take care of myself first. It took me a long time to realize that my health matters just as much as anyone else’s.”

Several participants expressed a desire to raise awareness about precocious menopause within their communities. Some had begun speaking openly about their experiences, aiming to break the stigma associated with early menopause. “I don’t want other women to feel as lost and alone as I did,” one participant explained. “If sharing my story helps even one person, then it’s worth it.”

**Table 2:** Thematic analysis – Women’s experiences of precocious menopause

Theme	Subtheme	Description	Example participant quote
Physical and Psychological Impact	Symptom Burden and Physical Changes	Participants reported disruptive physical symptoms (e.g., hot flashes, fatigue, sleep disturbances) that significantly affected daily life and long-term health.	“I never imagined feeling this overwhelmed by sudden heat and constant fatigue at such a young age. It has completely altered my daily routine.”
	Emotional and Mental Health Challenges	Women experienced deep emotional distress, including anxiety, depression, and loss of self-identity, often feeling isolated and overwhelmed by the unexpected onset of menopause.	“It feels like I’ve lost a part of who I am—my femininity, my future as I once envisioned it.”
Socio-Cultural Influences	Cultural Stigma and Societal Expectations	Societal norms linking womanhood to fertility led to stigma and feelings of inadequacy among participants, complicating their emotional adjustment to early menopause.	“In our society, a woman’s value is often measured by her ability to bear children. Experiencing menopause early feels like a betrayal of that role.”
	Family and Marital Dynamics	Family and marital relationships played a crucial role, with some participants facing pressure and judgment, while others benefited from supportive and understanding family dynamics.	“My husband and in-laws expected me to keep trying for children, even when my body was telling me otherwise.”
Coping Strategies and Adaptation	Reliance on Spiritual and Religious Beliefs	Many women turned to spirituality for comfort, viewing their experiences as a test of faith and finding solace in religious practices and community support.	“I’ve come to believe that this is a test of my strength and faith. Praying and reading the Quran helps me find peace.”
	Seeking Social and Peer Support	Peer support networks, both in-person and online, provided essential emotional and practical assistance, helping women feel less isolated and more resilient in facing their challenges.	“Talking to other women who understand exactly what I’m going through has been a lifeline. It reminds me I’m not alone in this journey.”
Personal Growth and Empowerment	Reframing Identity and Life Perspectives	The journey through precocious menopause spurred personal growth, prompting many women to redefine their identity and discover new aspects of themselves beyond traditional roles.	“I’ve learned to view this not as an end, but as a turning point in my life.”
	Resilience and Self-Advocacy	Despite the adversity, women demonstrated resilience by actively seeking information, advocating for improved care, and empowering themselves through community engagement.	“Before my diagnosis, I never questioned doctors. Now, I make sure to ask the right questions and advocate for myself in every medical appointment.”

This thematic analysis highlights the complex interplay of physical, emotional, socio-cultural, and institutional factors that shape the experiences of Egyptian women with precocious menopause. The insights gained from these themes provide a deeper understanding of the challenges faced by these women and underscore the need for comprehensive, culturally sensitive support systems and healthcare services.

## Discussion

This study explored the lived experiences of Egyptian women diagnosed with precocious menopause, providing rich insights into the physical, psychological, socio-cultural, and healthcare-related challenges they face. Our findings reveal a multifaceted experience that extends beyond the clinical manifestations of early ovarian failure, highlighting how personal identity, family dynamics, and cultural expectations interplay to shape women’s perceptions and coping mechanisms.

The physical and psychological impact of precocious menopause was profound among the participants. Many women described a sudden onset of disruptive symptoms such as hot flashes, fatigue, and sleep disturbances that not only interfered with daily activities but also raised concerns about long-term health consequences [23]. These findings are consistent with previous studies indicating that early menopause is associated with an increased risk of osteoporosis and cardiovascular disease [24]. Furthermore, the emotional distress characterized by feelings of grief, anxiety, and a diminished sense of femininity emerged as significant themes. The unexpected nature of precocious menopause led to a loss of identity, as many participants equated their value and womanhood with reproductive capability [25]. This sense of loss resonates with earlier research that has linked menopause with profound psychosocial changes and a redefinition of self-identity [26–28].

Socio-cultural factors played a pivotal role in shaping the experiences of these women. In the Egyptian context, where cultural norms place a high premium on fertility and motherhood, the diagnosis of precocious menopause often carried a stigma [29]. Many participants reported feeling judged by their families and communities, which further exacerbated their emotional turmoil. The pressure to conform to traditional expectations not only deepened the sense of isolation but also hindered open discussions about their condition. This aligns with studies from similar cultural settings that underscore the negative impact of societal expectations on women’s psychological well-being following reproductive health challenges [30, 31]. The narratives provided by our participants reveal that these societal pressures contribute to a feeling of inadequacy and loss, intensifying the psychological burden of precocious menopause.

Family dynamics emerged as another crucial factor influencing women’s experiences. While some participants described supportive relationships that provided emotional and practical assistance, others faced considerable pressure from family members who expected them to continue fulfilling traditional roles despite their altered reproductive status [32]. This divergence in family responses is critical, as supportive family environments can mitigate the adverse emotional effects of early menopause, whereas unsupportive or judgmental interactions may compound feelings of isolation and despair [33]. Our findings suggest that interventions aimed at educating family members about the nature of precocious menopause could play an essential role in fostering a more supportive home environment.

The study also highlighted the importance of coping strategies and adaptive mechanisms in managing the challenges of precocious menopause. Many women relied on spiritual and religious beliefs as a source of comfort and resilience. In a society where faith plays a central role in everyday life, viewing their experience as a test of strength allowed participants to find meaning and solace in their struggles. This reliance on spirituality is well-documented in the literature as a common coping mechanism among women facing chronic health conditions [34, 35]. Additionally, the formation of peer support networks—both in-person and online—provided a crucial outlet for sharing experiences, advice, and emotional support. These networks helped to break the isolation that many women felt and fostered a sense of solidarity and collective empowerment [36]. The role of peer support in improving psychological outcomes has been recognized in various studies, emphasizing the value of community-based approaches to health challenges [37].

Healthcare experiences and information barriers were significant themes in our study. Many participants reported feeling marginalized within the healthcare system, as healthcare providers often appeared unprepared to address the unique needs of younger women undergoing menopause [38]. The traditional association of menopause with older age meant that these women sometimes encountered dismissive attitudes, leading to delays in diagnosis and inadequate management of their symptoms. This finding is in line with previous research that highlights gaps in healthcare services for women with precocious menopause [39]. The lack of tailored care and clear communication contributed to a growing mistrust of healthcare providers and a sense of abandonment [40]. Enhancing the training of healthcare professionals to recognize and address the specific challenges faced by younger menopausal women could potentially improve patient outcomes and satisfaction [41].

Despite the substantial challenges, our study also revealed that the journey through precocious menopause can lead to significant personal growth and empowerment. Many women reported a transformative redefinition of their identities, moving from a focus on reproductive loss to embracing broader aspects of their self-worth [42, 43]. The process of reframing their identity was marked by increased resilience and a renewed determination to advocate for their health. Participants described how the adversity of early menopause compelled them to become more proactive, seeking out information and demanding better care [44]. This sense of empowerment is critical, as it not only improves individual well-being but also has the potential to drive broader social change by raising awareness about the condition [45].

### Implications of the study for practice and nursing

This study highlights the urgent need for nursing interventions that are responsive to the complex emotional, social, and cultural dimensions of precocious menopause. Nurses, as key healthcare providers, can play a pivotal role in improving care through the following targeted strategies:

#### Education and training

Nursing education should incorporate focused content on early menopause, including symptom management, psychosocial support, and cultural sensitivity. Continuing education programs can equip practicing nurses with skills in empathetic communication and holistic care for younger menopausal women.

#### Nurse-Led support initiatives

The establishment of nurse-led support groups within clinical or community settings can provide a confidential space for women to share experiences and access guidance. These programs should be patient-informed and address emotional resilience, stigma, and culturally relevant coping strategies.

#### Culturally competent counseling

Nurses should be trained to deliver counseling that acknowledges the influence of cultural norms, fertility-related stigma, and family expectations. Care plans should integrate patients’ spiritual and social values while encouraging informed decision-making.

#### Community engagement

Collaborating with religious and community leaders can help raise awareness and reduce stigma through public education campaigns, workshops, and outreach programs in accessible venues.

#### Policy and System-Level advocacy

Nurses can contribute to policy development by advocating for the inclusion of early menopause in national reproductive health agendas, expanding access to mental health support, and ensuring affordability of treatment options.

These strategies underscore the importance of a patient-centered and culturally responsive nursing approach to support women navigating the challenges of precocious menopause.

### Limitations of the study

While this study provides valuable insights into the experiences of Egyptian women with precocious menopause, it is not without limitations. First, the study utilized a qualitative research design, which, while effective in capturing in-depth personal narratives, limits the generalizability of the findings to a broader population. The sample size was relatively small, and all participants were recruited from specific healthcare facilities, which may not fully represent the diversity of experiences among Egyptian women from different geographical, socioeconomic, and educational backgrounds. Future studies incorporating larger and more diverse samples, including women from rural areas or different cultural backgrounds, could enhance the representativeness of the findings.

Second, self-reported data formed the primary source of information, which may introduce recall bias or social desirability bias, where participants may have provided responses that align with perceived societal expectations rather than their unfiltered experiences. Although efforts were made to create a safe and open discussion environment, some participants may have refrained from sharing highly personal or distressing details about their experiences.

Third, the study did not include perspectives from family members or healthcare providers, who play a significant role in shaping women’s experiences with precocious menopause. Including these perspectives in future research could provide a more comprehensive understanding of the social and medical dynamics surrounding early menopause. Additionally, a longitudinal approach could provide further insights into how women’s coping mechanisms, health outcomes, and perceptions evolve over time.

## Conclusion

This study provides an in-depth exploration of the lived experiences of Egyptian women diagnosed with precocious menopause, revealing a complex interplay of physical challenges, emotional distress, cultural expectations, and healthcare encounters. The findings highlight that beyond biological symptoms, early menopause profoundly disrupts women’s sense of identity, social belonging, and emotional well-being—particularly in contexts where fertility is central to perceptions of womanhood.

Cultural stigma, family pressure, and limited societal awareness often intensify women’s psychological burden and contribute to feelings of isolation. Yet, many participants demonstrated resilience through spirituality, peer support, and self-advocacy. Importantly, the study underscores the need for culturally sensitive, patient-centered nursing care that validates these experiences and responds to both their emotional and social dimensions.

The findings call for specific actions in nursing education and practice, including training on early menopause, nurse-led support initiatives, and community-based outreach to reduce stigma. Nurses are uniquely positioned to provide empathetic care, facilitate open dialogue, and advocate for systems that recognize the needs of younger women undergoing menopause.

Addressing precocious menopause requires a multidisciplinary, culturally informed approach that bridges clinical care with social understanding. This study contributes to filling the knowledge gap and provides a foundation for developing targeted interventions, policies, and educational strategies that improve the quality of life for women affected by early ovarian failure in Egypt and similar cultural contexts.

## Electronic supplementary material

Below is the link to the electronic supplementary material.

Supplementary Material 1

## Data Availability

The datasets generated during and/or analyzed during the current study are available from the corresponding author on reasonable request.
