# Supplementary material for: I’m still quite young: women’s lived experience of precocious or premature menopause: a qualitative study among Egyptian women
Source: BMC Nurs. 2025 May 28;24:612. doi: 10.1186/s12912-025-03133-6 (PMC12121103; doi:10.1186/s12912-025-03133-6)
Supplement: Supplementary file 1 — Supplementary Material 1 [file 12912_2025_3133_MOESM1_ESM.docx]

# Interview Guide: Lived Experiences of Egyptian Women with Precocious Menopause

Thank you for participating in this study. We aim to understand your experiences with precocious menopause and how it has affected different aspects of your life. There are no right or wrong answers—please feel free to share your thoughts and emotions openly. Your responses will remain confidential.

## 1. Initial Reactions & Emotional Response

Can you describe how you felt when you were first diagnosed with precocious menopause? What thoughts and emotions went through your mind?

## 2. Physical & Psychological Impact

What physical and emotional challenges have you experienced as a result of your condition? How have these symptoms affected your daily life and well-being?

## 3. Social & Cultural Influences

How has precocious menopause affected your relationships with family, friends, or your spouse/partner? Have you faced any societal or cultural challenges related to your condition?

## 4. Coping Strategies & Support Systems

What strategies or support systems (e.g., religious beliefs, family support, peer groups, healthcare professionals) have helped you cope with the challenges of precocious menopause?

## 5. Healthcare Experiences & Needs

Can you describe your interactions with healthcare providers regarding your diagnosis and treatment? What kind of support or information do you wish you had received?
